# What interventions are effective on reducing inequalities in maternal and child health in low- and middle-income settings? A systematic review

**DOI:** 10.1186/1471-2458-14-634

**Published:** 2014-06-21

**Authors:** Beibei Yuan, Mats Målqvist, Nadja Trygg, Xu Qian, Nawi Ng, Sarah Thomsen

**Affiliations:** 1 Division of Global Health (IHCAR), Department of Public Health, Karolinska Institutet, Nobels väg 9, SE-171 77, Solna Stockholm, Sweden; 2 Peking University China Center for Health Development Studies, Mailbox 505, Xue Yuan Road 38, Beijing 100191, China; 3 International Maternal and Child Health, Department of Women's and Children's Health, Uppsala University, University Hospital, Akademiska sjukhuset, Uppsala SE-751 85, Sweden; 4 School of Public Health, Fudan University, Shanghai 200032, China; 5 Unit of Epidemiology and Global Health, Department of Public Health and Clinical Medicine, Faculty of Medicine, Umeå University, 90187 Umeå, Sweden

## Abstract

**Background:**

The deadline for achieving Millennium Development Goals 4 and 5 is approaching, but inequalities between disadvantaged and other populations is a significant barrier for progress towards achieving these goals. This systematic review aims to collect evidence about the differential effects of interventions on different sociodemographic groups in order to identify interventions that were effective in reducing maternal or child health inequalities.

**Methods:**

We searched the PubMed, EMBASE and other relevant databases. The reference lists of included reviews were also screened to find more eligible studies. We included experimental or observational studies that assessed the effects of interventions on maternal and child health, but only studies that report quantitative inequality outcomes were finally included for analysis.

**Results:**

22 articles about the effectiveness of interventions on equity in maternal and child health were finally included. These studies covered five kinds of interventions: immunization campaigns, nutrition supplement programs, health care provision improvement interventions, demand side interventions, and mixed interventions. The outcome indicators covered all MDG 4 and three MDG 5 outcomes. None of the included studies looked at equity in maternal mortality, adolescent birth rate and unmet need for family planning. The included studies reported inequalities based on gender, income, education level or comprehensive socioeconomic status. Stronger or moderate evidence showed that all kinds of the included interventions may be more effective in improving maternal or child health for those from disadvantaged groups.

**Conclusion:**

Studies about the effectiveness of interventions on equity in maternal or child health are limited. The limited evidence showed that the interventions that were effective in reducing inequity included the improvement of health care delivery by outreach methods, using human resources in local areas or provided at the community level nearest to residents and the provision of financial or knowledge support to demand side.

## Background

Most low- and middle-income countries have made significant advances in reducing maternal and child mortality rates, even though these countries are still faced with serious inequalities in maternal and child health between different segments of the populations. For example, the overall maternal mortality ratio in China decreased from 64/100 000 in 1996 to 38/100 000 in 2008, but women in the poorest rural areas still had about 5 times higher risk of maternal mortality than women in urban areas until 2006 [1]. In Viet Nam the latest population census in 2009 showed that the under-five mortality rate decreased to 24/1000, as compared to 58/1000 in 1990 [2], but ethnic minorities still have a higher risk of under-five and neonatal mortality compared to the general population [3,4]. Ghana has not performed well in achieving the MDGs 4 and 5 and the populations in the poorest quintile still use less maternal and child health care services, including skilled care at birth, delivery in a health facility and use of modern contraceptives [5]. The deadline for achieving the Millennium Development Goals (MDGs) 4 and 5 is approaching fast, but the inequity between disadvantaged and other populations is a significant barrier for this progress.

Different kinds of interventions aimed at reducing maternal and child mortality rates have been implemented in these low- and middle-income countries. Some interventions try to improve maternal and child health by improving material circumstances where women or children live, such as provision of nutrition supplementation [6]; some interventions try to improve the delivery of maternal and children health services, such as promotion of immunization by outreach campaign [7], training traditional birth attendant [8], and upgrading health facility infrastructure and equipment for health care [9]; some interventions target demand sides and remove the physical, financial or other barriers to access to maternal or child health services, such as , subsidies for use of health services [10], community based information, education and communication interventions [11]. Depending on their target population, these interventions can be categorized into universal or targeted. Universal approaches aim at the whole population, and targeted interventions are aimed at specific groups, usually the disadvantaged [12]. Universal interventions may have differential effects in different segments of populations, and it is possible that an intervention that is intended to improve health in the overall population may widen inequalities if its benefits are concentrated among the better-off [13]. Whether the poor and other disadvantaged populations benefit less or more from health policies and interventions has also become a concern of policymakers and researchers when assessing the effectiveness of these health interventions [12]. Assessment of different effects in different population strata is also relevant for targeted interventions, as the effects might be different across population subgroups even within a disadvantaged group. For example, nutrition interventions targeting poor populations may have different effects on boys and girls [6].

Although there are systematic reviews evaluating effects of some implemented interventions on improving maternal and child health outcomes [14-17], we have not found any systematic reviews that analyze whether these interventions helped or hampered reducing inequalities in maternal and child health. We carried out this systematic review to explore if the interventions aimed to improve maternal and child health have different effects on different sociodemographic groups in order to identify which interventions were effective in reducing maternal or child health inequalities.

## Method

### Search strategy

We identified original studies in all languages by searching nine databases about health, social science and grey literature and dissertation databases, which included Cochrane Library (search date: 2014.04.01), PubMed (2014.04.02), Embase (2012.04.05), PsycINFO (2012.04.06), Global health (2012.04.06), Popline (2012.03.19), JSTOR (2012.03.29), ProQuest Dissertation & Theses Database (2014.04.03) and ISI proceeding (2012.03.21). All databases were searched from their earliest collecting date to the search date. Terms about maternal or child health, equity or disadvantaged populations, low- and middle-income countries and intervention studies were combined in the search strategy. We first designed the search strategy in PubMed (Additional file 1), and then translated this search strategy into the other electronic databases using the appropriate controlled vocabulary as applicable and free-text terms. We also examined references of relevant studies and reviews to identify additional relevant papers.

### Inclusion criteria

Inclusion criteria were discussed and agreed upon by all authors before starting this systematic review. We included experimental or observational study designs that assessed the effects of one or more kinds of interventions on maternal and child health, including randomized controlled trials, cluster randomized controlled trials, quasi-randomized controlled trials, controlled before-after studies, time series studies, before and after studies, cohort studies and case control studies.

We included all interventions designed to improve maternal and/or child health in any segment of the population (both universal interventions and targeted interventions) and/or those interventions specifically designed to reduce inequalities in maternal and/or child health exclusively in low and middle-income countries based on the World Bank List of Economies 2011 [18].

In terms of outcome measures, we only considered studies reporting the effects of interventions on official MDG 4 and 5 indicators, including under-five mortality rate, infant mortality rate, 1 year-old children immunized against measles, maternal mortality ratio, births attended by skilled health personnel, contraceptive use, adolescent birth rate, antenatal care coverage (at least one visit and at least four visits) and unmet need for family planning. Finally, only studies that report the inequalities outcomes were included. The inequalities outcomes could include quantitative descriptive difference for individuals or groups with different sociodemographic characteristics, absolute or relative concentration index, the slope or relative index of inequality [19], or any other measures used by authors for measuring change in inequalities after interventions. We used PROGRESS + categories as the sociodemographic indicators, which are: place of residence, race/ethnicity, occupation, gender, religion, education, socioeconomic status, social capital, age or disability [12].

### Study selection

Two review authors (BY and MM) independently scanned titles and abstracts of all articles obtained from the initial search to exclude those studies which were not evaluation studies about effectiveness of interventions to improve maternal and children health. Review authors (BY, MM and NT) then continued screening the kept titles and abstracts (all intervention studies) to only include those studies about effectiveness on disadvantaged populations or equity, which were further screened by reading the full texts in order to only retrieve those studies evaluating effects of interventions on equity. During the selection process, all review authors resolved any disagreements on inclusion through discussion.

### Data extraction and quality assessment

Key information such as intervention content, target population, study design, inequalities measure method, and maternal or children health outcome stratified by the sociodemographic characteristics specified in the PROGRESS criteria, was extracted from included full texts into an Excel file. The quality of each study was assessed by one review author (BY or NT) and checked by a second (MM or ST). We assessed study quality using a six-item checklist of quality criteria developed for the Effective Public Health Practice Project in Hamilton, Ontario [20]. In this checklist, six items include selection bias, study design, confounder control, blinding, data collection method and withdrawals and dropouts during study execution. Two review author firstly independently rated each item on a scale from “strong”, “moderate” to “weak”, and then jointly rated the methodology quality of whole study into “strong” (no weak rating for each item), “moderate” (one weak rating for each item) or “weak” (two or more weak ratings for each item). During this assessment process, all review authors resolved any disagreements on judgment through discussion.

### Data synthesis

We synthesized the effects of interventions on equity using a narrative method. The narrative summary can be used in systematic reviews when meta-analysis is not possible because the heterogeneity in interventions or outcome measures or any other reasons. Typically this method involves the ordering or chronicling of evidence to produce an account of it [21]. In this review, we firstly categorized and summarized the characteristics of included studies, including intervention content, outcome indicators, and study methodological quality. Because our aim is to identify which interventions were effective in reducing maternal or child health inequalities and we are not committed to synthesize the magnitude of inequalities at this stage. So in the analysis, for each kind of intervention and dimension of inequality, we summarized if the intervention was more effective in more advantaged groups (increased inequality); the intervention was more effective in more disadvantaged groups (decreased inequality); or there was no social gradient in the effectiveness of the intervention (no increase or decrease). If the change in inequality reported by authors was statistically significant, we categorized it into “Increase” or “Decrease”. Combined with the quality of studies, we analyzed if the conclusion was influenced by the studies’ methodological quality.

## Results

**Figure 1:**
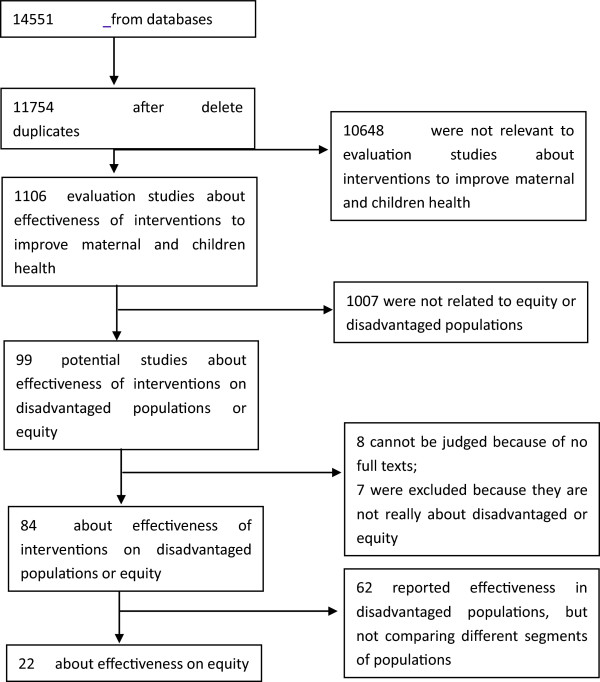
Study screening flow diagram.

We found 14,551 references from 9 databases, and 11,754 were left after deleting duplicates, in which 1,106 were evaluation studies about interventions to improve maternal and child health. Of the 1,106 references, only 99 were related to the effectiveness of interventions on disadvantaged populations or different effects between different segments of populations. After reading the full texts of these potentially relevant studies, we finally included 22 articles which had result about effects of interventions on different segments of populations (Figure 1). Those studies only reporting effectiveness in disadvantaged populations, but not comparing different segments of populations were reported in another systematic review [22].

Table 1 shows the research content of the included studies. We found effectiveness on inequalities in the following five kinds of interventions: immunization campaigns, nutrition supplement programs, health care provision improvement interventions, demand side interventions, and mixed interventions.

**Table 1:** Description of included studies

**Article and country of study**	**Intervention type**	**MDG outcome**	**Study design and overall quality**	**Inequality dimension**	**Inequality measurement and outcomes**
Aquino 2009 [23] Brazil	Interventions to improve provision of maternal and child health care services: Family Health Program (Strengthening community health service provision)	MDG 4: Infant and under-five mortality	Ecological study	Socioeconomic status	Fixed-Effects Models stratified by human development index of a district; Reductions in infant mortality rates measured by Risk Ratios after Family Health Program being more in lower Human development index group
			Moderate		
Baqui 2008 [24] India	Interventions to improve provision of maternal and child health care services: NGO facilitation of a government community-based MCH program	MDG 5: Skilled birth attendance and antenatal care visit	Controlled before and after study	Asset/wealth	Concentration indices (CI) calculated for intervention and comparison districts at baseline and endline for each outcome; The change of CI from baseline to endline in intervention districts being significant, but not in comparison districts
			Strong		
Benn 2010 [6] Guinea-Bissau	Nutrition supplements: Vitamin A	MDG 4: Infant and under-five mortality	Controlled trial	Gender	The differences in mortality between Vitamin A group and Placebo group, and stratification by sex; mortality rate ratios of vitamin A supplementation showing the supplementation tended to be beneficial in boys but not in girls
			Strong		
Bishai 2002 [25] Bangladesh	Expanding immunization coverage campaign: Intensive outreach visits by community health workers	MDG 4: Measles vaccination	Cohort study	Education, size of living room	Interactions between socioeconomic status indictors and a dummy variable for residence in intervention areas was added in probit regressions; The effects of parents’ education and living size being both lessened in intervention areas
			Strong		
Bishai 2003 [26] Bangladesh	Expanding immunization coverage campaign: Measles vaccine	MDG 4: Infant and under-five mortality	Cohort study	Socioeconomic status	Socio-economic differentials in mortality between the lowest and highest socioeconomic status (SES) quintiles in a cohort of vaccinated children and a control cohort; The mortality ratio of lowest SES to highest in the control population being significantly higher than it in the vaccinated population
			Strong		
Bishai 2005 [27] Nepal	Nutrition supplements: Vitamin A	MDG 4: Infant and under-five mortality	Controlled trial	Gender, caste and asset	The differences in mortality between boys and girls in Vitamin A group and placebo group; The difference in Vitamin A group being less than the difference in placebo group
			Strong		
Dyer 1996 [28] South Africa	Expanding immunization coverage campaign: National immunization campaign	MDG 4: Measles vaccination	Repeated cross-sectional study	Ethnicity	The differences vaccination coverage between pre- and post- campaign, and stratified for race; The difference in Asians being more than the differences in urban and rural blacks
			Weak		
Hatt 2007 [29] Indonesia	Interventions to improve provision of maternal and child health care services: A midwife in every village	MDG 5: Skilled birth attendance	Repeated cross-sectional study	Asset/wealth	Time-trend interactions with wealth quintile and urban/rural residence was added into logistic regression; The Odd Ratios showing that professional attendance in low socioeconomic status group increased more than those with higher socioeconomic status
			Weak		
Hotchkis 2011 [30] Nigeria, Uganda, Bangladesh and Indonesia	Interventions to improve provision of maternal and child health care services: Expansion of the private sector supply of modern contraceptives	MDG 5: Modern contraceptive prevalence Rate	Repeated cross-sectional study	Asset/wealth	Concentration indices differences between different survey rounds; concentration indices overall showing decrease trend
			Moderate		
Koenig 2001 [31] Bangladesh	Expanding immunization coverage campaign: Measles vaccine	MDG 4: Infant and under-five mortality	Cohort study	Gender, household dwelling size and education levels	Interactions between socioeconomic status indictors and vaccination status was added in proportional hazard regression; The decline in mortality risks with measles vaccination for those from smaller household dwelling size significantly exceeding that for those with bigger dwelling size, but no significant results for other two dimensions (gender and education level)
			Moderate		
Kozhimannil 2009 [32] Phillipines	Demand side interventions: Health insurance program	MDG 5: Antenatal care attendance	Repeated cross-sectional study	Asset, occupation and place of residence (rural/urban)	Stratified logistic analyses by urban/rural, employment status, and wealth; In women in rural areas, those in the lowest wealth tertile and those who are employed, the odds ratios being bigger and significant
			Moderate		
Masanja 2005 [33] Tanzania	Interventions to improve provision of maternal and child health care services: Integrated management of Childhood Illness	MDG 4: Measles vaccination	Controlled before and after study	Socioeconomic status	Concentration indices differences from 1999 to 2002 in both intervention and control groups; The difference in concentration indices in intervention group reducing more
			Moderate		
Nasreen 2003 [34] Bangladesh	Interventions to improve provision of maternal and child health care services: BRAC intervention (Providing preventive health, nutrition education and other primary care)	MDG 4: Infant and under-five mortality	Case control study	Education, occupation, economic condition, age	Stratified analysis for association between BRAC membership and neonatal death; For those mothers aged less and those fathers without wage, the relative risks of death being greater in intervention groups, and no significant influence from economic condition and parents’ literacy
			Strong		
Nielsen 2005 [35] Guinea-Bissau	Nutrition supplements: Vitamin A	MDG 4: Infant and under-five mortality	Controlled before and after study	Education levels, ethnicity, place of residence	Changes in mortality related to socio-economic factors for Vitamin A supplemented children compared with pre-wartime (no Vitamin A supplementation); the mortality risks being significantly reduced after war (Vitamin A supplemented) in the area pre-war disadvantage of families living and for those with low mother education level
			Moderate		
Pebley 1991 [36] Indiana	Mixed interventions: Combined health-care and nutrition service provision	MDG 4: Infant and under-five mortality	Controlled trial	Gender	The changes in male/female mortality ratios from before to during the intervention in different interventions and control groups; the male/female mortality ratios being increased more in intervention groups than change in control group
			Weak		
Sasaki 2011 [7] Zambia	Expanding immunization coverage campaign: Outreach immunization services	MDG 4: Measles vaccination	Repeated cross-sectional study	Distance to health facilities and income	Two logistic regression analysis before and after the introduction of outreach immunization services; Before the introduction of outreach services, longer distances to the service points being associated with lower measles immunization coverage, but not after the outreach services; no significant outcome for income
			Moderate		
Zeng 2011 [37] China	Nutrition supplements: Multiple micronutrients	MDG 4: Infant and under-five mortality	Controlled trial	Asset/wealth	Analysis for association between interventions and mortality outcome stratified by household wealth index; in the poorest group Iron/folic acid supplement significantly being related lower early neonate mortality, the association being not significant in wealthier group
			Strong		
Kamiya 2013 [38] Bangladesh	Mixed interventions: Safe Motherhood Promotion Project including demand side intervention (community mobilization through participatory approaches) and intervention to improve service provision	MDG 5: Skilled birth attendance and antenatal care visit	Controlled before and after study	Income	Interaction term, Income quartile × Project × Time was added in Difference-in-differences logistic regressions; Relative to the comparison site, significant increase in antenatal care for women belonging to lower income quartiles compared to those in the highest quartile in the project site, no significant outcome for skilled birth attendance
			Strong		
Quayyum 2013 [39] Bangladesh	Interventions to improve provision of maternal and child health care services: BRAC program’s the intervention on improving Maternal, Neonatal and Child Survival (IMN CS)	MDG 5: Skilled birth attendance and antenatal care visit	Controlled before and after study	Asset/wealth	The change in concentration index over time in both intervention and comparison areas; The concentration index being reduced more in the intervention areas
			Strong		
Amudhan 2013 [40] India	Mixed interventions: demand side interventions (conditional cash transfer scheme) and interventions to improve services provision (the strengthening of the primary health centre network)	MDG 5: Skilled birth attendance	Controlled before and after study	Socio-economic status: caste and education	Subgroup analysis by socioeconomic status on institutional delivery rate; institutional delivery among disadvantaged mothers increasing more compared with the increase among other groups
			Moderate		
Houweling 2013 [41] Indian	Demand side interventions: Participatory women’s group intervention	MDG 4: Infant and under-five mortality	Controlled before and after study	Socio-economic status: caste, land ownership, literacy, and asset ownership	Separate random effects logistic regression, for the most and less socio-economically marginalized groups; Odd Ratios showing that mortality reducing more in most marginalized populations than the reduction in less marginalized populations
			Strong		
Mosquera 2012 [42] Colombia	Interventions to improve provision of maternal and child health care services: Primary Health Care (PHC) strategy	MDG 4: Infant and under-5 mortality rate	Ecological study	Socio-economic status	Change in concentration indices for four child health outcomes from before to after implementation of intervention; The variation in the concentration index observed between the two periods being positive
			Weak		

The outcomes studied by the included studies cover all MDG 4 outcomes, but only three MDG 5 outcomes. The other MDG 5 outcomes, maternal mortality, adolescent birth rate, and unmet need for family planning were not reported. For the inequality dimension of outcomes, the included studies reported outcomes by gender, race or ethnicity, economic status, educational level and place of residence. The inequalities measure methods varied in different studies, and they can be roughly grouped into three categories: and the association analysis between intervention and outcomes being stratified by sociodemographic factors, interactions between sociodemographic indictors and intervention status being added in regression model, and the change in concentration index.

### Expanding immunization coverage campaign

Five studies, one published in 1996 and four published after 2000 (2002–2011), evaluated immunization expanding interventions in Bangladesh [25,26,31], South Africa [28] and Zambia [7]. The interventions aimed to increase measles immunization rates through outreach immunization services provided by community health workers [7,25] and widespread publicity to encourage and increase the availability of vaccinations [28]. Another two studies regarded measles vaccination as an intervention, and evaluated its effects on equity in under-five mortality rates [26,31]. The evidence from one strong study [25] and one moderate study [7] suggested that outreach immunization services may be more effective in improving measles immunization for those children whose mothers had lower educational backgrounds, those living in small rooms [25] and those living far from health facilities [7]. The same quality level of evidence showed that measles immunization could reduce the differences in child mortality between children living in different sizes of homes and between children with different socioeconomic status [26,31]. One weak study did not come to a clear conclusion on whether or not immunization publicity campaigns reduce inequality in measles immunization rates between different races [28] (Table 2).

**Table 2:** Effects of different interventions on inequalities

	**Expanding immunization coverage**	**Nutrition supplements**	**Interventions to improve health care provision**	**Demand side interventions**	**Mixed interventions**
**Measles immunization rate**	Education↓(++);		Socioeconomic status ↓(+)		
	Living area ↓(++);				
	Ethnicity ? (-);				
	Income ? (+);				
	Distance to health facilities↓(+)				
**Under-five children mortality rate**	Living area ↓ (+);	Gender↓	Socioeconomic status↓(-);		Gender↓(-)
	Socioeconomic status↓ (++)	(++);			
		Castes↓			
	Gender ? (+);	(++);			
	Education ? (+)	Asset ? (++)			
		Places of residence↓(+);			
		Education↓ (+);			
		Ethnicity ? (+)			
**Infant mortality rate**		Gender↑(++);	Socioeconomic status ↓(+,-);	Socioeconomic status↓(++)	Gender↓(-)
		Wealth↓(++)			
			Occupation↑(++);		
			Age↑(++);		
			Economic condition ? (++)		
			Education ? (++)		
**Antenatal care visit**			Wealth↓(++)	Places of residence↓(+)	Income↓(++)
				Asset↓(+)	
				Occupation↑(+)	
**Skilled births attendance**			Wealth↓(++, -)		Income ? (++)
					Socioeconomic status↓(+)
**Contraceptive use**			Wealth↓(+)		

↓Inequity was reduced, ↑Inequity was increased, ? No increase or decrease.

++Strong methodology quality; + Moderate methodology quality; - Week methodology quality.

### Nutrition supplements

Four studies published between 2005 and 2011 evaluated nutrition supplements on under-five children or neonatal mortality in China [37], Guinea-Bissau [6,35] and Nepal [27]. In two studies the supplements targeted children aged 6 to 60 months [27,35]. One study targeted low birth weight neonates [8], and another targeted pregnant women until delivery [37]. The nutrition supplement interventions included Vitamin A supplements [6,27,35], multiple micronutrients, and iron/folic acid supplementation [37]. The evidence from the two studies with strong quality was inconsistent: in Nepal universal supplementation with vitamin A narrowed differentials in child deaths across gender [27], but in low birth weight neonates of Guinea-Bissau vitamin A supplementation had a significant negative effect on neonatal mortality in girls [6]. Vitamin A supplementation also proved to be effective in narrowing mortality differentials among castes, places of residence and mothers’ education levels [27,35]. Zeng’s study [37] with strong quality found that both multiple micronutrients and iron/folic acid supplementation had positive effects on neonatal mortality among the poorest in socioeconomic status, but had no significant effects in wealthier households.

### Interventions to improve provision of maternal and child health care services

Eight studies published from 2003 to 2013 assessed the effects of different interventions to improve maternal and child health care provision in Bangladesh [30,34,39], Columbia [42], Brazil [23], India [24], Indonesia [29,30], Nigeria [30], Tanzania [33] and Uganda [30]. The interventions varied, including:

Integrated Management of Childhood Illness (IMCI), was implemented in Tanzania. In this intervention, the prevention and treatment of common childhood illnesses were combined into simple guidelines and messages for use in primary health facilities and communities. The study with moderate quality suggested that IMCI reduced inequalities in measles immunization rates between different socioeconomic quintiles [33].

The Family health program in Brazil used multi-professional teams working under the principles of comprehensive care to provide permanent and systematic follow-up of a given number of families residing in a circumscribed area. Priority actions include promotion, prevention, and care for mothers and children. The moderate quality level of evidence showed this program in Brazil had greater effect in reducing infant mortality rate in municipalities with higher infant mortality rates and lower human development index scores [23].

The BRAC health program in Bangladesh provided preventive health and nutrition education, as well as immunization, family planning, pregnancy and reproductive health related care and basic curative services, which were delivered by voluntary community health workers selected from the women’s credit group members by means of regular household visits. This intervention was found to have more effects in advantaged populations, including elder mothers’ children and children whose father were waged laborers, because BRAC provided less attention to adolescent and households with lower income during implementation [34]. As a key element of BRAC health program, the selected community health workers providing intensive home-based maternal and newborn care was specifically evaluated by an controlled before and after study [39], and the evaluation showed that intervention resulted in the pro-poor increase in the utilization of antenatal care and trained attendants for home delivery.

Primay health care strategy in Columbia included a core program “Salud a su Casa”. The program worked in the network of first-level facilities and public hospitals, and twelve hundred families were assigned to a multidisciplinary basic health care team. Basic health care teams either provided intra or extramural services. A study [27] found this intervention contributed the reductions of the inequality associated with socioeconomic status in child health outcomes, but this study provided weak evidence because it just analyzed the concentration curves change before and after the strategy implementation without control groups.

Training and posting a midwife in every village with specific responsibility for pregnancy, delivery and postpartum care was the intervention content of the Indonesian village midwife program which aimed to increase the proportion of deliveries managed by trained professionals, particularly among poor rural populations. This program resulted in increased professional attendance, with the greatest increases occurring among the poorest two income quintiles [29].

Collaboration between NGOs and the government on community-based maternal and child health care was implemented in Uttar Pradesh, India. This partnership emphasized use of existing infrastructure, training of community-based workers, home visits to promote behavior change, complete geographical coverage during pregnancy and postnatal period, creating support for community-based workers by recruiting community volunteers and strengthening supportive supervision. This intervention was proved by a strong study to be effective in reducing inequality in antenatal care check-up and medically trained birth attendant between families with different asset quintiles [24].

Private sector supply of modern contraceptives was expanded in Nigeria, Uganda, Bangladesh and Indonesia in order to improve the availability of reproductive health care supplies and services. The approach was criticized for potentially leading to increases in disparities in health care services utilization. But a study across these four countries evaluated this expansion and found that inequalities based on socioeconomic status showed a decrease trend in the four countries [30].

### Demand side interventions

Two studies published between 2009 and 2013 evaluated the impact of demand side interventions on antenatal care visits and neonatal mortality rate in Philippines [32] and India [41].

### Health insurance program

One study published in 2009 evaluated if the National health insurance program (PhilHealth program) in Philippines was effective in improving access to prenatal and delivery care. Subgroup analyses in this moderate quality study showed that this health insurance program could reduce inequalities; with the strongest impacts among women in rural areas, those in the lowest wealth tertile and those who were employed [32].

Community-based participatory intervention was a kind of participatory women’s group intervention, in which the participatory groups met regularly, guided by a facilitator to discuss and identify maternal and newborn health problems. This intervention could help increase people’s awareness and demand for maternal health services and then improve women’s health-care seeking behaviors. This kind of intervention was also verified by strong evidence [41] to have stronger effectiveness on reduction of neonatal mortality rate among the most marginalized women.

### Mixed interventions

Three studies published from 1990 to 2013 assessed how the mixed interventions improve child health and maternal health care utilization in India [36,40] and Bangladesh [38]. The contents of mixed interventions are varied, including:

### Combined health-care and nutrition service provision

One study [36] published in 1990 assessed the effect of combinations of nutrition and health services provision interventions in India. This study included three intervention groups (one group with nutritional supplementation, one group with a health-care program and the other group with integration of nutrition with the health-care program) and one control group, and looked at which intervention was more successful in reducing excess female mortality. There was a substantial reduction in the gap between male and female mortality in all three treatment groups, while there was little change in the control villages. Among the treatment groups, the biggest reduction in excess female mortality was in the combined-services group, followed by the nutritional-services group and the health-care group. However, this study was rated as weak in methodological quality because it lacked reporting on confounding factors and follow-up rates.

Safe Motherhoo Promotion Project (SMPP) was conducted in the Narsingdi district of Bangladesh. This intervention included community mobilization through participatory approaches and strengthening of organizational and personnel capacities for delivering emergency obstetric care (EmOC) at district and sub-district level hospitals. A well-conducted controlled before and after study [38] provided strong evidence that this intervention reduced income inequalities in access to antenatal care, but has no significant impact on skilled birth attendants.

### Combined conditional cash transfer (CCT) and the strengthening of the primary health centre (PHC) network

Primary health centres (PHCs) being selected to provide obstetric care with additional resources and a conditional cash transfer scheme were implemented in a staggered manner in some areas of India. One study [40] evaluated the impact of each intervention and the combination of them on inequality in use of skilled birth delivery, and this moderate evidence showed that the combined interventions, especially strengthening of the PHC network added to CCT, could result in a bigger increase in institutional delivery among the disadvantaged than among others.

## Discussion and conclusion

### Principal findings

This review comprehensively collected and assessed evidence about effects of interventions on maternal and child health in low- and middle-income countries, and focused on assessing the available evidence about the role of these interventions in tackling health inequalities. Overall, we found that the number of original studies assessing the effectiveness of health interventions on inequality is still limited. During the screening process, although we found 1,106 evaluation studies about effectiveness of interventions aimed at improving maternal and children health, only 22 reported quantitatively the effects on different segments of populations.

We found evidence from current limited original studies that immunization outreach campaigns can reduce inequalities in immunization rates across different levels of education, living area and distance to health facilities, and that measles immunization could help reduce socioeconomic inequalities in child mortality.

Well-conducted studies also found that nutrition supplementation could be more effective in reducing infant mortality among different kinds of disadvantaged groups. But the evidence with respect to differential effects of vitamin A supplement by gender are not consistent: for reducing mortality of children aged 6–60 months girls benefitted more in Nepal [27], but vitamin A supplementation was harmful for low-birth weight neonate girls in Guinea-Bissau [6]. The success of vitamin A supplement in reducing inequality is because public health interventions relying on door-to-door distribution of nutrition essentially overcome intra-household gender biases that could otherwise be prejudiced against girls. Failure of vitamin A supplementation in reducing inequality for neonates may be because of initial differences in vitamin A status (boys have lower retinol levels in their cord blood than do girls) or a negative interaction between immunomodulating micronutrients and the DTP vaccine [6].

Though the contents of health care provision interventions included in our review were different from each other, most of them were successful in reducing the inequalities in maternal health care utilization or child health outcomes, mainly because these interventions in several Asian and African countries all emphasized nutrition or primary health care provision at the community level. A review of equity in maternal, newborn, and child health interventions in 54 countries also found that interventions that are carried out at the community level tended to be more equitable than those that are usually conducted in health facilities [43]. But intervention content including primary health care provision at the community level did not mean it would result in improved equity. For example, in the BRAC program of Bangladesh advantaged populations benefited more because in the process of policy implementation not enough attention was paid to adolescent and low-income populations [34].

Not like interventions aimed to improve delivery of immunization, nutrition or health services, another kind of intervention is to address the demand side barriers to maternal and children health services. For example health insurance and conditional cash transfer can reduce the economic barrier to health care accessibility; community participatory intervention targeted the problem of disadvantaged population usually lacking awareness on health problems or importance of health care. The evidence collected by this review verified the disadvantaged population benefited more from this kind of interventions. The reasons are that these interventions could achieve or tried to ensure the uptake of the intervention (insurance coverage or participatory meeting) being similar among different kinds of populations; and when the uptake was universal, the effective interventions usually had stronger effects on the disadvantaged populations [41].

Some interventions were combination of the above interventions, and the evaluation of these combined interventions could remind us that both provision improvement and demand side inventions are indispensable for reducing inequality. For example Amudhan’ study [40] found that the impact of strengthening of the PHC network added to CCT on reduction of inequality was bigger than the impact of only CCT. This result showed that demand side subsidy works better if the delivery system is accessible. Kozhimannil’ study [32] mainly found that PhilHealth insurance program contributed to the reduction of inequalities, but this study also mentioned that another provision improvement invention at the same period (exposure to midwife clinics) was not associated with significant changes in the use of prenatal care.

### Strengths and weaknesses of the available evidence

There are prospective experimental designs or observational studies with control groups for most kinds of interventions we included. Based on the six-item quality criteria, only four studies were rated as weak in methodology. A challenge for non-experimental studies is how to differentiate the effects of different interventions implemented simultaneously, because maternal and children health can be influenced by many kinds of interventions, and in developing countries there are usually many kinds of development or health interventions at the same time. Despite this, authors often did not report other interventions or contextual factors that might have influenced the effects of the intervention.

Information about how the interventions were implemented would help policy-makers and readers better understand how interventions could be effective in reducing inequity. Though most of the included studies discussed the possible reasons for how interventions influenced inequalities in maternal or child health, few studies described the implementation process of interventions in detail. Process evaluations would improve readers’ understandings of which components were actually implemented, and help policymakers understand the challenges and benefits of various components in an intervention [12].

### Strengths and weaknesses of the review

We attempted to obtain both published and unpublished relevant studies, and we also tried to include a wide range of study designs in order not to miss useful evidence, because for some community interventions targeting maternal or child health it is difficult to conduct experimental studies. However, it is possible that we have not identified all relevant interventions for which the effects on inequalities have been explored, since we only found five categories of interventions from so many interventions having been implemented to improve maternal and child health in low- and middle-income countries.

This review aims to find which interventions were effective in reducing maternal or child health inequalities, so we focused more on the direction of the interventions’ effect on inequality (i.e. if the intervention decreased or increased inequality), but not the extent of inequality changed by the interventions. Besides, it is also difficult to synthesize the quantitative outcome related to equity because the inequality outcome was measured differently in different studies: some used the change in concentration index after intervention [30,33]; some added the interaction between sociodemographic variables and intervention variables in a multivariate analysis model [29,31]; some used the difference in change of outcome with interventions between different social groups [27,35,37].

Incorporating the heterogeneity in intervention, study design, study quality and study outcomes were challenges for this systematic review. To manage this we referred to Thomas’s analysis method [13] in his systematic review to do descriptive synthesis in order to incorporate the interventions, the study outcomes, study quality and sociodemographic stratified for equity analysis. Thomas developed a novel graphic method to synthesize and display the balance of evidence to illustrate the possible different effects of the interventions. We only illustrated them in a table because for each intervention, each outcome and each dimension of equity, only one or two studies were found in this systematic review.

### Implications for policy and future research

From this evidence, we find that the first most important characteristics of these interventions in reducing inequality is that they were all related to basic health care for pregnant women or children, including immunization and other preventive health services, treatment of common illnesses, nutrition education or direct nutrition supplements. Secondly, in terms of health care or nutrition provision approaches, these interventions focused on outreach methods (home visit and follow-up by health workers), making use of human resources in local areas, and provision of services at the community level nearest to residents. This kind of approach can overcome many barriers for disadvantaged populations to access health services and improve health status, like economic barriers, long distance to health facilities and limited knowledge about the importance of basic health care. Thirdly, another kind of inventions is to provide financial or knowledge support to demand side in order to reduce the above mentioned barriers to access health services. Consequently, for public interventions to reduce inequalities in maternal and child health, basic health care relying on door-to-door distribution by community health workers or direct support to demanders should be the critical elements. Even if these public health interventions took a universal coverage approach, they may still improve equity.

We have identified several gaps in the evidence base about interventions to reduce inequalities in maternal or child health. In particular, studies about the differential effects of interventions on maternal or child health by sociodemographic indicators are limited. Given that many universal or targeted interventions for achieving MDG 4 or 5 have been implemented in low- and middle-income countries in recent years, more subgroup analyses should be conducted when evaluating these interventions in order to shed light on effects on inequalities [44]. Even for evaluations of targeted interventions, subgroup analysis to look at effects on inequalities can provide evidence about how to better design targeting methods used by these interventions. For example, the interventions targeting less-developed areas may not result in the really disadvantaged populations in these areas gaining more. Moreover, the contextual and implementation process information is very important for explaining how interventions influenced inequities and providing implication for policy design. However, we found that in the current evidence there was a lack of contextual information and implementation process information for interventions, which should be included in future research.

## Competing interests

We declare that we have no conflicts of interest.

## Authors’ contributions

All authors have contributed to the production of this manuscript. All review authors discussed and contributed to conceptualization of this review and development of review protocol. BY, MM, NT and ST applied the inclusion criteria, data extraction and quality assessment. BY prepared the first draft and all other authors commented on and revised it. All authors read and approved the final manuscript.

## Pre-publication history

The pre-publication history for this paper can be accessed here:

http://www.biomedcentral.com/1471-2458/14/634/prepub

## Supplementary Material

Additional file 1 Search Strategy used in PubMed. Click here for file
