# Supplementary material for: What interventions are effective on reducing inequalities in maternal and child health in low- and middle-income settings? A systematic review
Source: BMC Public Health. 2014 Jun 21;14:634. doi: 10.1186/1471-2458-14-634 (PMC4083351; doi:10.1186/1471-2458-14-634)
Supplement: Additional file 1 — Search Strategy used in PubMed. [file 1471-2458-14-634-S1.pdf]

Appendix 1 Search Strategy used in PubMed

#1: Reproductive health services[MH] OR Maternal Welfare[MH] OR Maternal Mortality[MH] OR Contraception[MH] OR Pregnancy[MH] OR Abortion, Induced[MH] OR Abortion, Spontaneous[MH] OR Prenatal Care[MH] OR Delivery, Obstetric[MH] OR Postnatal Care[MH] OR Family Planning Services[MH] OR "MDG 5"[TIAB] OR "millennium development goal five"[TIAB] OR "millennium development goal 5"[TIAB] OR "maternal healthcare" [TIAB] OR "maternal health"[TIAB] OR "maternal service"[TIAB] OR "maternal services"[TIAB] OR "maternal mortality"[TIAB] OR "maternal mortalities"[TIAB] OR "maternal morbidity"[TIAB] OR "reproductive health"[TIAB] OR "reproductive service"[TIAB] OR "reproductive services"[TIAB] OR contraception[TIAB] OR "birth control"[TIAB] OR "fertilization control"[TIAB] OR "family planning"[TIAB] OR "planned pregnancies"[TIAB] OR pregnancy[TIAB] OR gestation[TIAB] OR abortion[TIAB] OR "pre-natal care"[TIAB] OR "pre-natal visit"[TIAB] OR "pre-natal visits" [TIAB] OR "prenatal care"[TIAB] OR "prenatal visit"[TIAB] OR "prenatal visits"[TIAB] OR "antenatal care"[TIAB] OR "antenatal visits"[TIAB] OR "antenatal visit"[TIAB] OR "obstetric delivery"[TIAB] OR "obstetric deliveries"[TIAB] OR "delivery care"[TIAB] OR "facility-based delivery"[TIAB] OR "skilled birth attendance"[TIAB] OR "institutional delivery"[TIAB] OR "postnatal care"[TIAB] OR "post-natal care"[TIAB] OR "postpartum program"[TIAB] OR "postpartum programs"[TIAB] OR "safe motherhood"[TIAB] OR "traditional birth attendant"[TIAB] OR "traditional birth attendants"[TIAB] OR "direct obstetric case fatality rate"[TIAB] OR "adolescent birth"[TIAB] OR "obstetric admissions"[TIAB] OR "gynecological admissions"[TIAB]

#2: Pediatrics[MH] OR Child Mortality[MH] OR Infant Mortality[MH] OR Perinatal Mortality[MH] OR Neonatal Mortality[MH] OR Stillbirth[MH] OR Premature Birth[MH] OR Prematurity[MH] OR Asphyxia Neonatorum[MH] OR Low Birth Weight[MH] OR Malnutrition[MH] OR Congenital Abnormalities[MH] OR Breast Feeding[MH] OR Immunization[MH] OR Vaccination[MH] OR "MDG 4"[TIAB] OR "millennium development goal four"[TIAB] OR "millennium development goal 4"[TIAB] OR pediatrics[TIAB] OR "child health"[TIAB] OR "child mortality"[TIAB] OR "child deaths"[TIAB] OR "child death"[TIAB] OR "under-five mortality"[TIAB] OR "under-five death"[TIAB] OR "infant mortality"[TIAB] OR "infant deaths"[TIAB] OR "infant death"[TIAB] OR "neonatal mortality"[TIAB] OR "neonatal deaths"[TIAB] OR "neonatal death"[TIAB] OR "newborn mortality"[TIAB] OR "newborn deaths"[TIAB] OR "newborn death"[TIAB] OR "perinatal mortality"[TIAB] OR "child survival"[TIAB] OR "newborn survival"[TIAB] OR "perinatal outcomes"[TIAB] OR "neonatal outcomes"[TIAB] OR stillborn[TIAB] OR premature[TIAB] OR "birth asphyxia"[TIAB] OR "asphyxia neonatorum"[TIAB] OR "umbilical infection"[TIAB] OR intra-partum[TIAB] OR malformation[TIAB] OR "breast feeding"[TIAB] OR immunization[TIAB] OR vaccination[TIAB] OR "IMCI"[TIAB] OR "PMTCT"[TIAB] OR "KMC"[TIAB] OR "kangaroo mother care"[TIAB] OR skin-to-skin[TIAB] OR "low birth weight"[TIAB] OR malnutrition[TIAB] OR "congenital abnormalities"[TIAB]

#3: Healthcare Disparities[MH] OR Health Status Disparities[MH] OR Universal Coverage[MH] OR Vulnerable Populations[MH] OR Poverty[MH] OR Rural Population[MH] OR Residential Mobility[MH] OR Emigration and Immigration[MH] OR Minority Groups[MH] OR Religion[MH] OR Ethnic Groups[MH] OR Adolescent[MH]

OR Social Class[MH] OR Socioeconomic Factors[MH] OR Insurance Coverage[MH] OR Gender[MH] OR equity[TIAB] OR inequity[TIAB] OR inequities[TIAB] OR equality[TIAB] OR inequality[TIAB] OR inequalities[TIAB] OR disparity[TIAB] OR disparities[TIAB] OR “universal coverage”[TIAB] OR “disadvantaged group”[TIAB] OR “disadvantaged groups”[TIAB] OR “disadvantaged population”[TIAB] OR “disadvantaged populations”[TIAB] OR “vulnerable population”[TIAB] OR “vulnerable populations”[TIAB] OR “sensitive population”[TIAB] OR “sensitive populations”[TIAB] OR poor[TIAB] OR poverty[TIAB] OR indigene[TIAB] OR indigent[TIAB] OR indigency[TIAB] OR self-employed[TIAB] OR “informal sector”[TIAB] OR “informal sectors”[TIAB] OR low-income[TIAB] OR unemployed[TIAB] OR “income distribution”[TIAB] OR slum[TIAB] OR rural[TIAB] OR urban[TIAB] OR “agricultural worker”[TIAB] OR “agricultural workers”[TIAB] OR “agricultural labor”[TIAB] OR “agricultural labors”[TIAB] OR farmer[TIAB] OR farmers[TIAB] OR farmworker[TIAB] OR farmworkers[TIAB] OR migration[TIAB] OR migrations[TIAB] OR migrant[TIAB] OR migrants[TIAB] OR emigrant[TIAB] OR emigrants[TIAB] OR immigrant[TIAB] OR immigrants[TIAB] OR minority[TIAB] OR minorities[TIAB] OR ethnicity[TIAB] OR ethnic[TIAB] OR religion[TIAB] OR religious[TIAB] OR tribal[TIAB] OR scheduled[TIAB] OR hindu[TIAB] OR islam[TIAB] OR muslim[TIAB] OR muslims[TIAB] OR Christian[TIAB] OR Christianity[TIAB] OR adolescent[TIAB] OR adolescents[TIAB] OR youth[TIAB] OR youths[TIAB] OR teen[TIAB] OR teens[TIAB] OR teenager[TIAB] OR teenagers[TIAB] OR “social class”[TIAB] OR “social classes”[TIAB] OR caste[TIAB] OR castes[TIAB] OR socioeconomic[TIAB] OR “social norms”[TIAB] OR education[TIAB] OR discriminatory[TIAB] OR discrimination[TIAB] OR uninsured[TIAB] OR insured[TIAB] OR “insurance status”[TIAB] OR “insurance coverage”[TIAB] OR “health insurance”[TIAB] OR homeless[TIAB] OR "HIV positive"[TIAB] OR "western area"[TIAB] OR "western areas"[TIAB] OR disability[TIAB] OR handicapped[TIAB] OR disabled[TIAB] OR boys[TIAB] OR girls[TIAB] OR “remote area”[TIAB] OR “remote areas”[TIAB] OR marginalisation[TIAB]

#4: Developing Countries[MH] OR Asia[MH] OR Africa[MH] OR Latin America[MH] OR South America[MH] OR Kenya[MH] OR Rwanda[MH] OR Sudan[MH] OR Tanzania[MH] OR Uganda[MH] OR Malawi[MH] OR Mozambique[MH] OR “South Africa”[MH] OR Zambia[MH] OR Zimbabwe[MH] OR Benin[MH] OR “Burkina Faso”[MH] OR Ghana[MH] OR Mali[MH] OR Niger[MH] OR Nigeria[MH] OR Senegal[MH] OR Algeria[MH] OR Egypt[MH] OR Libya[MH] OR Tajikistan[MH] OR Cambodia[MH] OR Indonesia[MH] OR Laos[MH] OR Malaysia[MH] OR Philippines[MH] OR Thailand[MH] OR Vietnam[MH] OR Bangladesh[MH] OR India[MH] OR China[MH] OR Brazil[MH] OR Chile[MH] OR Colombia[MH] OR Peru[MH] OR Mexico[MH] OR Nicaragua[MH] OR Haiti[MH] OR “low-income setting”[TIAB] OR “low-income settings”[TIAB] OR “low-income countries”[TIAB] OR “middle-income setting”[TIAB] OR “middle-income settings”[TIAB] OR “middle-income countries”[TIAB] OR “low income setting”[TIAB] OR “low income settings”[TIAB] OR “low income countries”[TIAB] OR “middle income setting”[TIAB] OR “middle income settings”[TIAB] OR “middle income countries”[TIAB] OR “transition economy”[TIAB] OR “transition economies”[TIAB] OR “economically disadvantaged”[TIAB] OR “less favored areas”[TIAB] OR “developing world”[TIAB] OR “GDP”[TIAB] OR “developing country”[TIAB] OR “developing countries ” [TIAB] OR “under-developed country”[TIAB] OR “under-developed countries”[TIAB] OR “less-developed country”[TIAB] OR “less-developed

countries”[TIAB] OR “less-developed nation”[TIAB] OR “less-developed nations”[TIAB] OR “third-world country”[TIAB] OR “third-world countries”[TIAB] OR Africa[TIAB] OR African[TIAB] OR Asia[TIAB] OR Asian[TIAB] OR “Latin America”[TIAB] OR “South America”[TIAB] OR Kenya[TIAB] OR Rwanda[TIAB] OR Sudan[TIAB] OR Tanzania[TIAB] OR Uganda[TIAB] OR Malawi[TIAB] OR Mozambique[TIAB] OR “South Africa”[TIAB] OR Zambia[TIAB] OR Zimbabwe[TIAB] OR Benin[TIAB] OR “Burkina Faso”[TIAB] OR Ghana[TIAB] OR Mali[TIAB] OR Niger[TIAB] OR Nigeria[TIAB] OR Senegal[TIAB] OR Algeria[TIAB] OR Egypt[TIAB] OR Libya[TIAB] OR Tajikistan OR Cambodia[TIAB] OR Indonesia[TIAB] OR Laos[TIAB] OR Malaysia[TIAB] OR Philippines[TIAB] OR Thailand[TIAB] OR Vietnam[TIAB] OR Bangladesh[TIAB] OR India[TIAB] OR China[TIAB] OR Brazil[TIAB] OR Chile[TIAB] OR Colombia[TIAB] OR Peru[TIAB] OR Nicaragua[TIAB] OR Haiti[TIAB]

#5: Intervention Studies[MH] OR Evaluation Studies as Topic[MH] OR Random Allocation[MH] OR Evaluation Studies[PT] OR Randomized Controlled Trial[PT] OR Controlled Clinical Trial[PT] OR Comparative Study[PT] OR impact[TIAB] OR impacts[TIAB] OR assess[TIAB] OR assessment[TIAB] OR evaluation[TIAB] OR evaluate[TIAB] OR appraisal[TIAB] OR appraise[TIAB] OR effectiveness[TIAB] OR effect[TIAB] OR effects[TIAB] OR “controlled trial”[TIAB] OR “controlled trials”[TIAB] OR cohort[TIAB] OR “time series”[TIAB] OR “case control”[TIAB] OR “before and after”[TIAB] OR "pre test"[TIAB] OR pretest[TIAB] OR posttest[TIAB] OR "post test"[TIAB] OR experiment[TIAB] OR experiments[TIAB]

#6: intervention[TIAB] OR interventions[TIAB] OR program[TIAB] OR programs[TIAB] OR programme[TIAB] OR programmes[TIAB] OR project[TIAB] OR “health policy”[TIAB] OR “health policies”[TIAB] OR “health promotion”[TIAB]

#7: (#1) OR #2

#8: ((((#3) AND #4) AND #5) AND #6) AND #7
